# Multisensory stimulation in advanced dementia: Insights from a case study

**DOI:** 10.1002/alz.090742

**Published:** 2025-01-09

**Authors:** Savvina Chrysostomou, Antonia Tziannarou, Andrea Hadjiloizou, Sotiria Moza

**Affiliations:** ^1^ Noesis Cognitive Center & Tech Solutions Ltd, Nicosia Cyprus; ^2^ Materia Group, Nicosia Cyprus

## Abstract

Background A 92‐year‐old retired seamstress, born in 1932, with 12 years of education, had been residing in a long‐term care facility since 2019, following a fall and hip fracture. Post‐admission, her cognitive function gradually declined and she did not participate in residential home activities. This study explores the outcomes of an 8‐month, multisensory remediation program. Methods The individual participated in bi‐weekly, multisensory, group therapy sessions spanning eight months from April to December 2023. The stimuli comprised a blend of visual, tactile, and auditory elements, such as constructive wooden puzzles, knitting wool, sensory balls, kinetic sand, exposure to fabric and clothing, classical music songs, language engagement through discussions, and reminiscence activities. Results Over the course of 8 months, the individual demonstrated qualitative improvements, including active participation in dialogues with neuropsychologists, expressing preferences for stimuli, and displaying increased engagement and willingness to attend sessions. Clinic personnel also noted positive changes in mood, cooperation, interest in interactive activities within the clinic, and enhanced language abilities. These improvements led to the patient relocating to a more team‐oriented table in the common area. As a result of these observed positive changes in the clinical profile, the Mini Mental State Examination (MMSE) was administered in December 2023, yielding a score of 6 out of 30 (points achieved in, naming, reading, orientation and following instructions). Discussion Multisensory therapy may show promise in regard to language and communication skills in elderly individuals with advanced dementia. These positive outcomes may contribute to the patients’ overall well‐being and serve as valuable indicators for treatment evaluation. The study is ongoing to explore long‐term effects and broader implications.

